# Relationships Between Teacher Feedback and Student Motivation: A Comparison Between Male and Female Students

**DOI:** 10.3389/fpsyg.2021.679575

**Published:** 2021-08-30

**Authors:** Wenjuan Guo, Wenye Zhou

**Affiliations:** ^1^School of Education, Shanghai Jiao Tong University, Shanghai, China; ^2^Institute of Curriculum and Instruction, East China Normal University, Shanghai, China

**Keywords:** teacher feedback, gender differences, student motivation, language learning, Chinese students

## Abstract

This study aimed to investigate gender differences in teacher feedback and students’ motivation in learning and their relationship patterns. In total, 1,082 secondary students in China (538 male and 544 female students) participated in this study. The results of MANOVAs suggested that language teachers provided less directive feedback but more criticism to male than female students. Male students reported less intrinsic motivation, extrinsic motivation and test anxiety than female students. The results of two-group structural equation modeling indicated that both male and female students’ motivation was best predicted by teachers’ scaffolding feedback and praise. Verification feedback had a negative correlation with female students’ extrinsic motivation and no significant correlation with male students’ motivation. Directive feedback had a negative correlation with male students’ intrinsic motivation and a positive correlation with female students’ extrinsic motivation. Further, teacher criticism only had a negative correlation with female students’ intrinsic motivation. Implications for future research as well as suggestions for teachers on how to improve male and female students’ motivation are discussed.

## Introduction

The vital role of motivation in students’ academic achievement and life-long learning beyond school has been well documented (Zimmerman, 2013; Mega et al., 2014; Guo, 2020). Motivation is defined as the sum of the need for achievement, the probability of success, the incentive values related to task fulfillment and the incentives to avoid failure (Dörnyei and Ushioda, 2013). However, the literature indicates that students’ motivation declines over age and is one of the main causes of their learning problems in school (Yeung et al., 2011). Therefore, increasing the level of motivation is pivotal for promoting students’ academic success. Further, the social cognitive theory suggests that student motivation is context specific and could be influenced by social environment (Bandura, 2011). Teacher feedback, which is conceptualized as the information offered by the teacher concerning aspects of student performance (Hattie and Timperley, 2007), is one of the social factors that have strong impact on student learning (Pereira et al., 2016).

However, despite the amount of the literature on teacher feedback, most have focused on the role of one or two types of teacher feedback (e.g., Lipnevich and Smith, 2008; Haimovitz and Corpus, 2011), and little is known about how various types of teacher feedback relate to motivation. Additionally, owing to social priming and gender-role stereotypes (Huang, 2013; Plante et al., 2013), the relationship between teacher feedback and student motivation may differ between male and female students, which was little examined. Thus, to bridge these research gaps, this study aimed to investigate gender differences in teacher feedback, students’ motivation and their relationships. Findings of this study would contribute to the literature by providing researchers as well as educators with useful insights of the role of different types of teacher feedback on male and female students’ motivation.

### Teacher Feedback

Teacher feedback has always been conceived from the cognitivist perspective as information about the weaknesses and strengths of students’ academic performance and how it can be improved (Hattie and Timperley, 2007). Though feedback plays a vital role in student learning, it has long been viewed by teachers as a tough, challenging and burdensome work (Carless and Winstone, 2020). The quality of feedback was perceived more positively by teachers than by students (Van der Kleij, 2019).

Based on the *functions of different types of teacher feedback* (i.e., verification, directive, scaffolding and motivational functions; Bangert-Drowns et al., 1991), five types were identified: *verification feedback*, *directive feedback*, *scaffolding feedback*, *teacher praise* and *teacher criticism* (Guo, 2017). *Verification feedback* refers to teachers’ dichotomous judgment of students’ academic performance by affirming it as being either correct or incorrect, or to the first providing marks, grades or rankings to the latter. *Directive feedback* refers to teachers providing direct answers or solutions to questions. *Scaffolding feedback* refers to a series of hints/prompts provided by the teacher for guiding students to independently generate correct answers to problems. *Teacher praise* refers to the act of commending the value of students’ learning attitudes, processes or outcomes. *Teacher criticism* refers to the act of providing negative responses to students’ learning attitudes, processes or outcomes.

Owing to social priming and gender-role stereotypes, generally, females are expected to perform better than males in language learning (Plante et al., 2013). For instance, research indicated that female students tend to use more self-regulated learning strategies and have higher motivation in language (Carr et al., 2016). Therefore, language teachers may provide more positive and less negative feedback to female than male students (Guo, 2017). Nevertheless, little was known about whether language teachers are affected by gender-role stereotypes and may provide varied feedback types when referring to either a male or a female student; thus, this study sought to diminish the gap in knowledge in this topic.

### Student Motivation

Motivation has been considered as a positive predictor of students’ academic achievement (Zimmerman, 2013; Mega et al., 2014). Moreover, the *self-regulated learning theory* suggests that – among various components of student motivation – *intrinsic motivation*, *extrinsic motivation*, *self-efficacy* and *test anxiety* are important aspects and integrating factors of self-regulated learning (Pintrich et al., 1991), and that these play an essential part in student achievement and commitment (Zimmerman, 2013); thus, this study will focus on such factors, which are described herein: *intrinsic motivation* refers to the degree to which learners engage in learning owing to curiosity or self-interest, reflecting the potential of human nature and our inherent tendency to learn (Pintrich et al., 1991; Yeung et al., 2011). *Extrinsic motivation* refers to the degree to which learners engage in learning owing to their desire for external rewards (Pintrich et al., 1991). *Self-efficacy* refers to personal judgments regarding one’s capabilities to attain designated goals, which can affect one’s choice of, effort and resilience applied in learning activities (Zimmerman, 2000; Yeung et al., 2011). *Test anxiety* refers to negative thoughts, affect and physiological arousal before taking a test (Pintrich et al., 1991).

Generally speaking, female students tend to have stronger motivations towards and abilities in first language learning: for instance, studies showed that female students, compared to male students, had higher intrinsic motivation and self-efficacy (Yeung et al., 2011; McGeown et al., 2012), and that they had higher extrinsic motivation (D’Lima et al., 2014). In addition, female students showed being able to control their test anxiety with more efficacy – by seeking help from peers and positive thinking – than their male counterparts (Kao et al., 2017). However, most previous studies were conducted in the context of foreign language learning, so little is known about these gender differences regarding student motivation in the context of first language learning, thereby limiting the possibility of generalizing research findings that relate to gender differences in studies on students’ motivation.

### Relationships Between Teacher Feedback and Student Motivation

Generally, the literature confirms that teacher feedback can effectively influence student motivation (e.g., Pereira et al., 2016; Guo and Wei, 2019; Guo et al., 2019; Van der Kleij, 2019; Lou and Noels, 2020). Scaffolding feedback may be the most positive type, as it was shown to influence student learning by facilitating the promotion of student independent learning (Guo and Wei, 2019; Guo et al., 2019). For instance, Guo and Wei (2019) found that teachers’ scaffolding feedback requires students to generate answers by themselves, and that such behavior can effectively promote students’ intrinsic and extrinsic motivation and self-efficacy, since scaffolding feedback allows students more autonomy and enhances positive teacher-student relationships, which was found to promote student motivation and academic performance effectively (Poulou, 2020). Moreover, studies suggested that teachers’ sincere and specific praise can reinforce students’ desired learning behaviors and significantly increase their extrinsic motivation and self-efficacy (Haimovitz and Corpus, 2011; Guo et al., 2019).

Contrastingly, teacher feedback can also be ineffective or even detrimental for student motivation (Lou and Noels, 2020). A study showed that verification feedback can exert negative effects on students’ motivation because they tend to redirect students’ focus from addressing problems to competing with peers, or please the teacher or their parents (Lipnevich and Smith, 2008). Further, directive feedback was found to develop students’ teacher dependency and decrease their intrinsic motivation and self-efficacy (Guo et al., 2019). Additionally, teacher criticism as a type of negative feedback was also found to decrease students’ intrinsic motivation and self-efficacy (Atlas et al., 2004; Thompson et al., 2020).

Notwithstanding, although there is a large body of research on teacher feedback and student motivation, little is known about gender differences and its relationship with and between these two variables in the context of first language learning. Owing to gender-role stereotypes and their influence on motivation-related beliefs (Carr et al., 2016; Moè and Putwain, 2020), there may exist gender differences in such relationships. For instance, even with the same feedback (either positive or negative), female students, compared to male, may feel more efficacious (Guo, 2017). Thus, further insight is warranted on gender differences regarding relationships between teacher feedback and student motivation.

### Research Questions

Based on the abovementioned theoretical and empirical descriptions, the following research questions were posited:

1. Are there differences in how male and female students perceived their language teachers’ feedback?
2. Are there differences in students’ motivation in language learning between male and female students?
3. Are there gender differences in the relationships between students’ perceptions of teacher feedback and their motivation in language learning?

## Materials and Methods

### Participants

This study’s sample comprised secondary students from the mainland of China. In total, 1,121students participated in this study, and 1,082 students [97%, 538 males (49.7%) and 544 females (50.3%)], aged between 15 and 17 years (*M* = 16.21, *SD* = 0.43), completed the questionnaire. Participants in this study came from two secondary schools in Shanghai, which were randomly selected by the author. To make sure that the study sample is more representative, the two different type of schools were recruited. One school is located in urban area and is equipped with adequate learning resources, such as high-tech learning devices and online tutorial courses, whereas the other one is located in suburban area and has very limited learning resources.

### Measures

#### Student Perceptions of Teacher Feedback

Teacher feedback was measured through a questionnaire created by Guo (2017) to measure students’ perceptions on how frequently their Chinese language teachers provided various types of feedback. There are five types of teacher feedback in the questionnaire: *verification feedback, directive feedback, scaffolding feedback, teacher praise* and *teacher criticism*. The questionnaire consists of 22 items, all of which are rated on a 5-point Likert scale (1 = *never* and 5 = *always*). Sample items and descriptive and internal consistencies for all scales are shown in Table 1.

**Table 1:** Sample items, internal consistency coefficients and descriptive statistics for the variables of students’ perceptions of teacher feedback measured in this study.

	Cronbach’s *α*	Sample item (no. of items in the scale)	*M*	*SD*
Verification feedback	0.75	My teacher gives a score or grade on our quiz (4)	5.23	0.812
Directive feedback	0.80	My teacher directly tells me the correct answer when I get an answer wrong in the classroom (4)	4.70	0.991
Scaffolding feedback	0.79	My teacher helps me solve problems by offering some hints or cues (5)	5.11	0.883
Teacher praise	0.88	My teacher praises or encourages me when I perform better than before (5)	4.72	1.105
Teacher criticism	0.85	My teacher criticizes or punishes me when I fail in exams (4)	3.82	1.216

#### Student Motivation

The Motivated Strategies for Leaning Questionnaire (Pintrich et al., 1991) was utilized, which is a measure designed to evaluate students’ motivation. It consists of four sections: *intrinsic motivation, extrinsic motivation, self-efficacy* and *test anxiet*y, each with 5 items. All items are rated on a 7-point Likert scale (1 = *strongly disagree* and 7 = *strongly agree*). Sample items and descriptive and internal consistencies for all scales are shown in Table 2.

**Table 2:** Sample items, internal consistency coefficients and descriptive statistics for the variables of students’ self-regulated learning measured in this study.

Scales	Cronbach’s *α*	Sample item (no. of items in the scale)	*M*	*SD*
Intrinsic motivation	0.88	I prefer course material that arouses my curiosity, even if it is difficult to learn (5)	4.89	1.26
Extrinsic motivation	0.86	Getting a good grade in this class is the most satisfying thing for me right now (5)	5.17	1.29
Self-efficacy	0.92	I’m certain I can master the skills being taught in this class (5)	4.80	1.26
Test anxiety	0.92	I feel my heart beating fast when I take an exam (5)	4.23	1.64

### Procedures

#### Data Collection

All students volunteered to participate this study and completed the questionnaire. Before data collection, the author sought permission from the school principal and teachers for the conduction of the study, and all participants provided informed consent, received a brief set of directions to help in their responses to the questionnaire and were told that confidentiality would be ensured and collected data would be exclusively utilized for research purposes. Generally, participants took about 10 min to complete the whole questionnaire, and these were immediately collected after their completion.

#### Data Analyses

Three sets of analyses were conducted. First, to ensure that both the students’ perceptions of teacher feedback measures and student motivational measures were equivalent between male and female students, we conducted a series of two-group confirmatory factor analyses (CFA) using the Mplus 7. Specifically, we examined the configural, metric, scalar and residual invariance, and factor variance and covariance of the two measures for males and females separately. Second, to examine gender differences in teacher feedback and students’ motivation, we computed two separate MANOVAs – while controlling for the class – using the SPSS 23 software. Third, we computed two-group structural equation modeling (SEM) using Mplus 7 to examine the gender differences in the relationship between teacher feedback and students’ motivation.

## Results

### Measurement of Invariance

As demonstrated in Table 3, for students’ perceptions of teacher feedback measures, each of the six invariance models had a good fit (RMSEAs < 0.039; CFIs > 0.934; TLIs > 0.925; and SRMRs < 0.059), and for student motivation measures, each of the six invariance models also had a good model fit (RMSEAs < 0.045; CFIs > 0.970; TLIs > 0.963; and SRMRs < 0.051). In the two measures and each invariance comparison, the decrease in CFI and TLI values was less than 0.01. Thus, for the two measures, there was strong evidence for the equality of loadings, intercepts, residuals, factor variance and covariances between male and female students.

**Table 3:** Measurement invariance tests for students’ perceptions of teacher feedback measures and SRL measures.

Model and invariance level	Over fit indexes						Model comparison	Comparative fit indexes			
	*χ* ^2^	*df*	RMSEA	CFI	TLI	SRMR		Δ*χ*^2^	Δ *df*	Δ CFI	Δ TLI
*Students’ perceptions of teacher feedback measures*											
1. Configural	865^*^	461	0.039	0.936	0.925	0.054					
2. Metric	888^*^	480	0.035	0.934	0.931	0.052	2 vs. 1	23	19	0.002	0.006
3. Scalar	851^*^	488	0.033	0.942	0.936	0.055	3 vs. 2	37	8	0.008	0.005
4. Residual	882^*^	512	0.031	0.944	0.939	0.057	4 vs. 3	31	24	0.002	0.003
5. Factor variance	890^*^	536	0.030	0.945	0.943	0.058	5 vs. 4	18	24	0.001	0.004
6. Factor covariance	921^*^	555	0.030	0.946	0.943	0.059	6 vs. 5	31	19	0.001	0.000
*Motivational measures*											
1. Configural	583^*^	284	0.041	0.974	0.966	0.045					
2. Metric	607^*^	300	0.040	0.974	0.966	0.048	2 vs. 1	24	16	0.000	0.003
3. Scalar	668^*^	316	0.042	0.970	0.963	0.049	3 vs. 2	61	16	0.004	0.001
4. Residual	687^*^	336	0.040	0.970	0.966	0.049	4 vs. 3	19	20	0.000	0.000
5. Factor variance	709^*^	359	0.043	0.971	0.968	0.050	5 vs. 4	22	23	0.001	0.002
6. Factor covariance	730^*^	378	0.045	0.973	0.972	0.051	6 vs. 5	21	19	0.002	0.004

*At each step numbered in each section in the sequence of invariance tests, all earlier constraints remain in place.*

* *p < 0.001*.

### Confirmatory Factor Analyses

Two CFAs were computed to examine the measurement model of students’ perceptions of teacher feedback and student motivation measures. Results suggested that the measurement model showed a good fit for the data in the students’ perceptions of teacher feedback measures (*χ*^2^ = 638.094; *df* = 223; *p* < 0.0001; RMSEA = 0.037; 90% CI [0.034,0.041]; CFI = 0.942; TLI = 0.927; and SRMR = 0.051). All factor loadings and correlations of the students’ perceptions of teacher feedback measures were significant (*β*s = 0.38–0.88; *r*s = 0.26–0.75; *p*s < 0.0001). The measurement model also showed a good fit for the data in the student motivation measure (*χ*^2^ = 372.428; *df* = 142; *p* < 0.0001; RMSEA = 0.036; 90% CI [0.031,0.040]; CFI = 0.973; TLI = 0.963; and SRMR = 0.042). All factor loadings and correlations of the student motivation measure were significant (*β*s = 0.64–0.92; *r*s = 0.13–0.72; *p* < 0.0001).

### Gender Differences in Students’ Perceptions of Teacher Feedback

**Figure 1:**
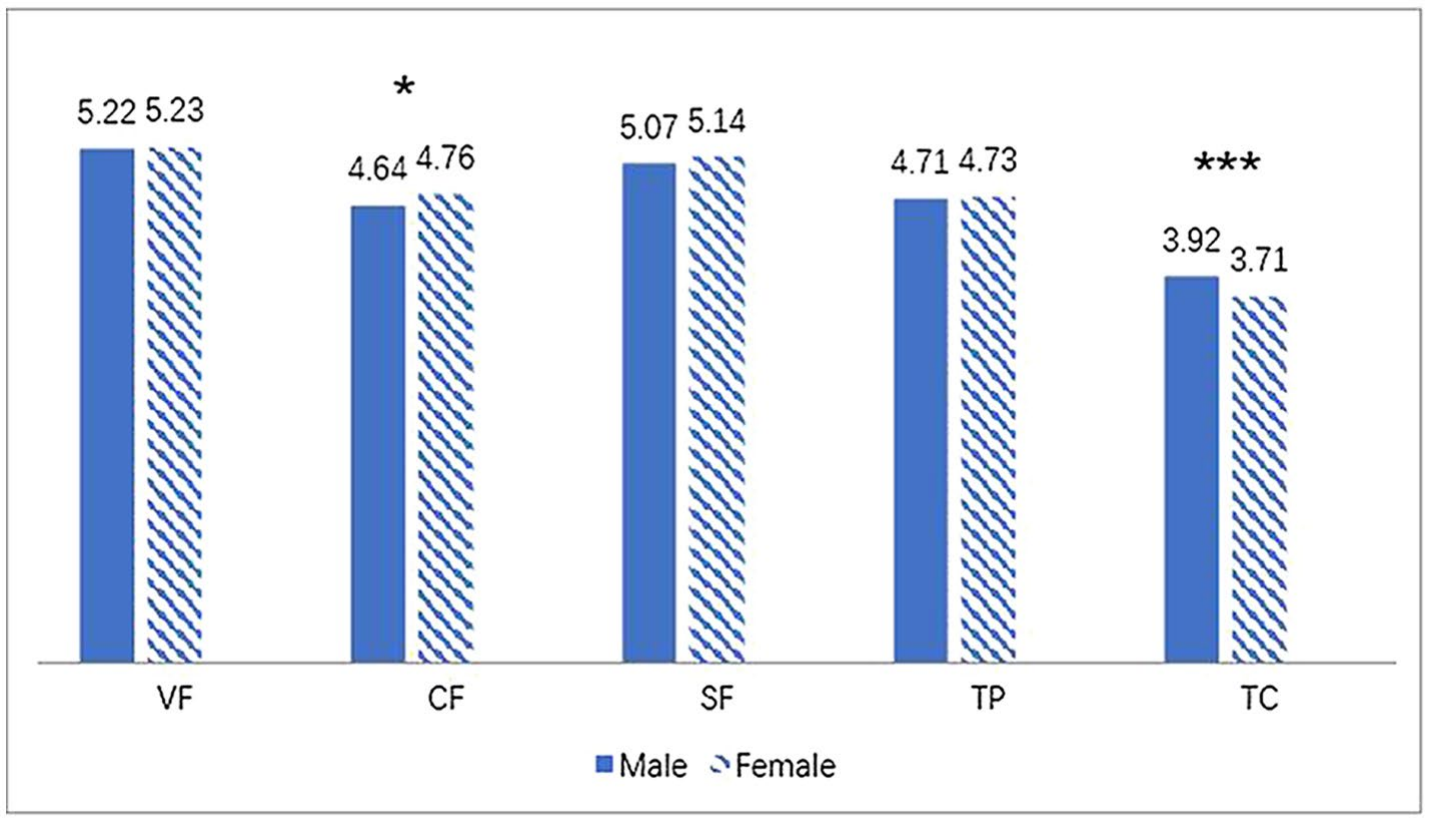
Language teachers’ feedback for male and female students. ^*^p < 0.05 and ^***^p < 0.001. VF, verification feedback; CF, corrective feedback; SF, scaffolding feedback; TP, teacher praise and TC, teacher criticism.

As shown in Figure 1, the results of the MANOVA indicated that male students perceived that their teachers offered less directive feedbacks [*F* (1, 1,080) = 4.340; *p* < 0.05; *η*^2^ = 0.004] and more criticism [*F* (1, 1,080) = 7.939; *p* < 0.001; *η*^2^ = 0.007] to males than females did. No significant differences were found in the other three types of teacher feedback (*p*s > 0.05).

### Gender Differences in Students’ Motivation

**Figure 2:**
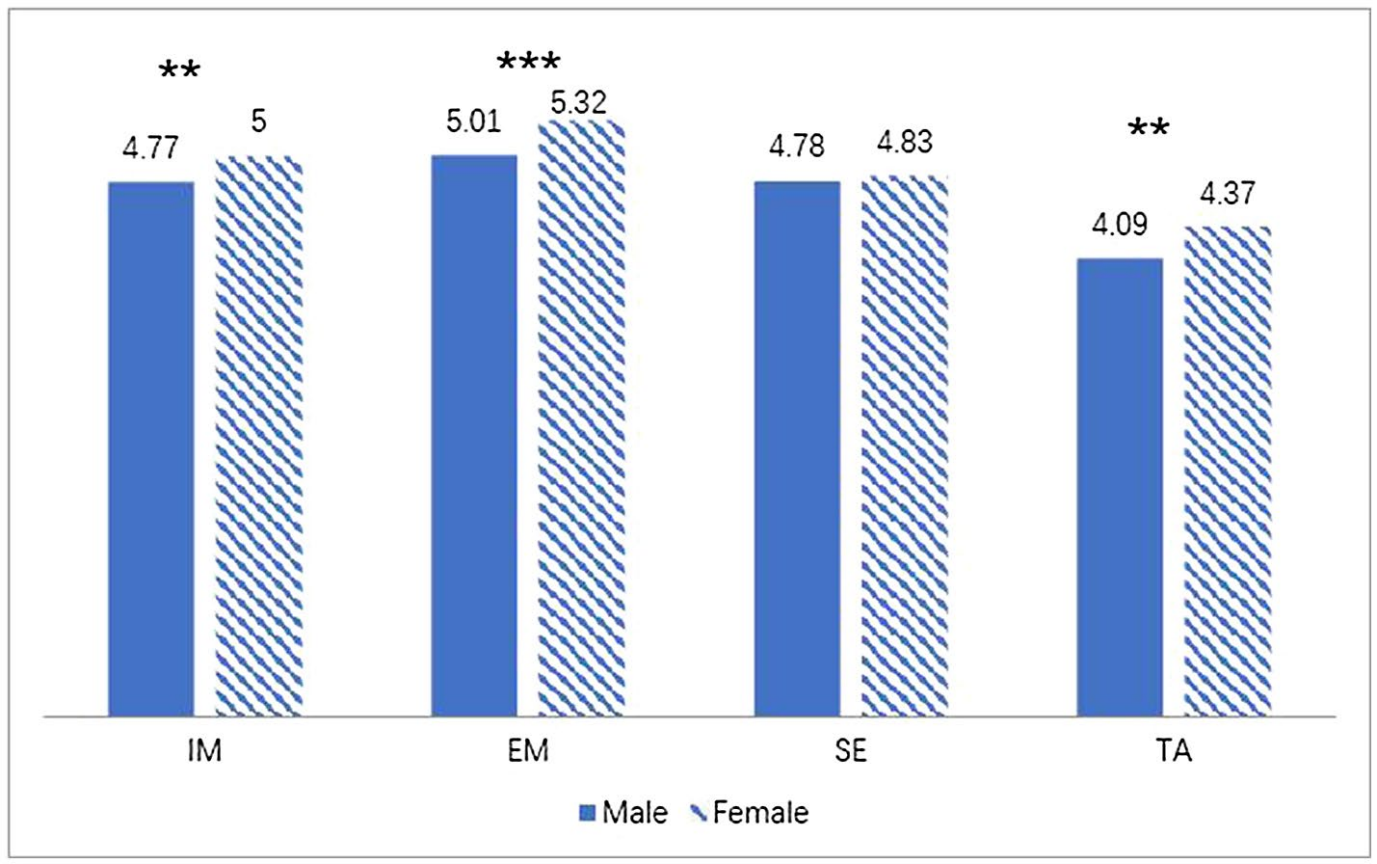
Gender differences in students’ motivation in language. ^**^p < 0.01 and ^***^p < 0.001. IM, intrinsic motivation; EM, extrinsic motivation; SE, self-efficacy and TA, test anxiety.

As shown in Figure 2, the results of the MANOVA suggested that, compared to female students, males reported lower levels of intrinsic motivation [*F* (1, 1,080) = 9.683; *p* < 0.01; *η*^2^ = 0.009], extrinsic motivation [*F* (1, 1,080) = 15.244; *p* < 0.001; *η*^2^ = 0.014] and test anxiety [*F* (1, 1,080) = 7.713; *p* < 0.05; *η*^2^ = 0.007]. No significant difference was found in self-efficacy (*p* > 0.05).

### Gender Differences in the Relationship Between Students’ Perceptions of Teacher Feedback and Student Motivation

As given in Table 4, zero-order correlations were initially performed to examine the relationship between teacher feedback and student motivation. Verification feedback showed significant correlations with both male and female students for all components of student motivation (*r*s = 0.10 to.15; *p*s < 0.05), except for test anxiety (*p*s > 0.05). Directive and scaffolding feedback and teacher praise had significant correlations with both male and female students for all components of student motivation (*r*s = 0.04 to.30; *p*s < 0.05). Teacher criticism had significant correlations with both male and female students for all components of student motivation (*r*s = 0.05 to.24; *p*s < 0.05), except for self-efficacy with male students, and intrinsic motivation with female students (*p*s > 0.05).

**Table 4:** Zero-order correlations among all variables of students’ perceptions of teacher feedback and male and female students’ motivation.

Scales	VF	CF	SF	TP	TC	IM	EM	SE	TA
VF	–	0.47^***^	0.57^***^	0.53^***^	0.25^***^	0.14^***^	0.16^***^	0.19^***^	0.04
CF	0.46^***^	–	0.43^***^	0.38^***^	0.25^***^	0.12^**^	0.17^***^	0.13^**^	0.11^*^
SF	0.55^***^	0.43^***^	–	0.65^***^	0.26^***^	0.17^***^	0.25^***^	0.22^***^	0.10^*^
TP	0.48^***^	0.34^***^	0.64^***^	–	0.44^***^	0.25^***^	0.30^***^	0.29^***^	0.14^**^
TC	0.21^***^	0.26^***^	0.28^***^	0.46^***^	–	−0.02	0.18^***^	0.10^*^	0.24^***^
IM	0.10^*^	0.05^***^	0.18^***^	0.26^***^	0.10^*^	–	0.46^***^	0.60^***^	0.05
EM	0.15^**^	0.10^***^	0.19^***^	0.25^***^	0.12^**^	0.48^***^	–	0.47^***^	0.32^***^
SE	0.11^**^	0.04^***^	0.16^***^	0.22^***^	0.05	0.71^***^	0.52^***^	–	0.00
TA	0.05	0.05^**^	0.11^**^	0.15^**^	0.21^***^	0.14^***^	0.38^***^	0.15^***^	–

*The lower half of the triangle is the correlations between teacher feedback and male students’ SRL strategy use in language; and the upper half of the triangle is the correlations between teacher feedback; and female students’ SRL strategy use in language*.

* *p < 0.05;*

** *p < 0.01;*

*** *p < 0.001.*

To further examine gender differences in the relationship between teacher feedback and student motivation, a two-group SEM model was computed. The two-group SEM model provided an adequate fit with the data (*χ*^2^ = 2330.684; *df* = 1758; *p* < 0.0001; RMSEA = 0.028; 90% CI [0.025,0.031]; CFI = 0.911; TLI = 0.904; and SRMR = 0.061).

**Figure 3:**
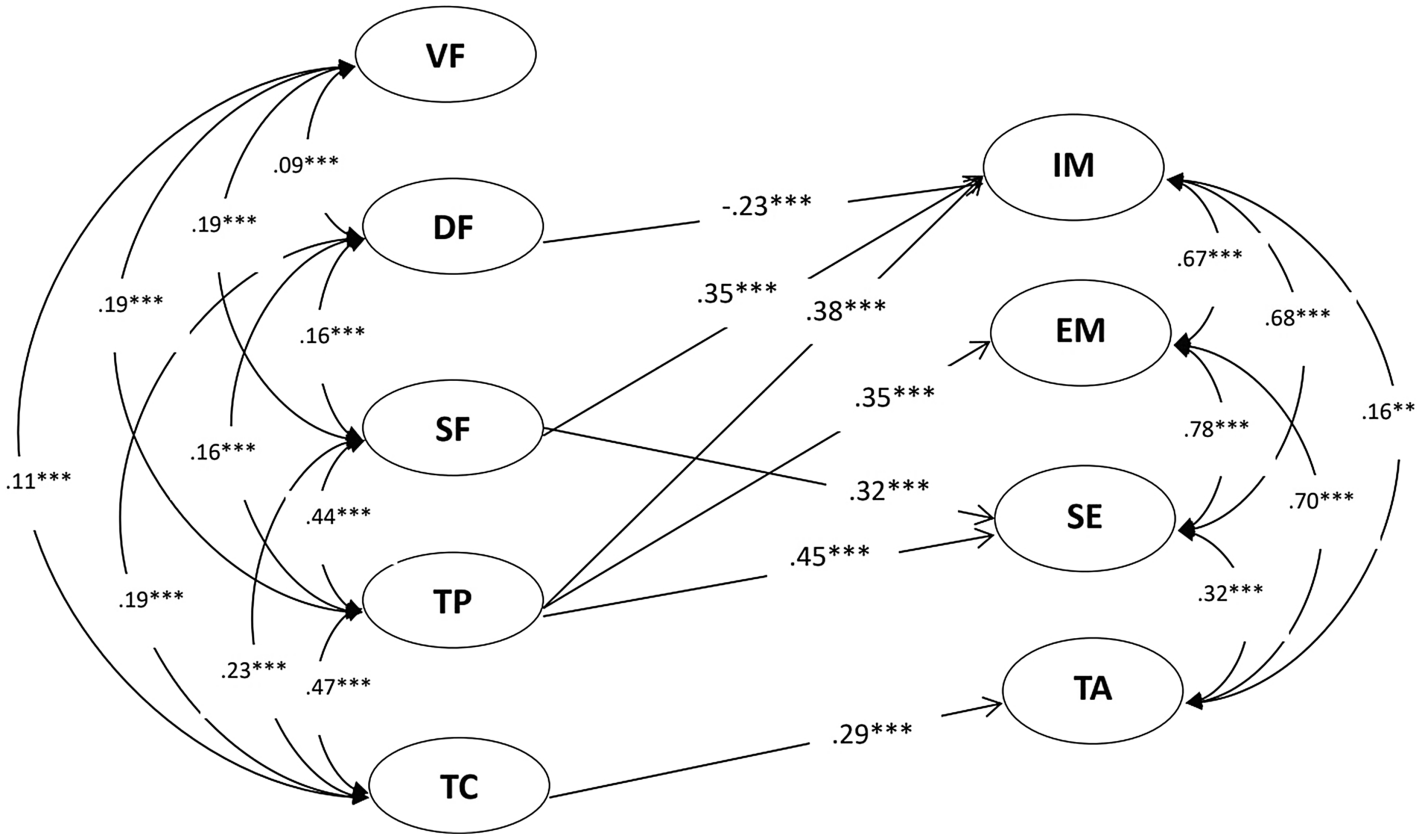
Relationships between male students’ perceptions of teacher feedback and their motivation in language. ^*^p < 0.05, ^**^p < 0.01 and ^***^p < 0.001. VF, verification feedback; DF, directive feedback; SF, scaffolding feedback; TP, teacher praise; TC, teacher criticism; IM, intrinsic motivation; EM, extrinsic motivation; SE, self-efficacy and TA, test anxiety.

As depicted in Figure 3, for male students, results indicated that verification feedback had no significant relationship with any components of motivation (*p*s > 0.05); directive feedback only had a negative relationship with intrinsic motivation (*r* = −0.23; *p* < 0.001); scaffolding feedback had positive relationships with intrinsic motivation and self-efficacy (*r*s = 0.32 to.35; *p* < 0.001); teachers’ praise had positive relationships with intrinsic and extrinsic motivation and self-efficacy (*r*s = 0.35 to.45; *p* < 0.001); and teachers’ criticism only had a positive relationship with test anxiety (*r* = 0.29; *p* < 0.001).

**Figure 4:**
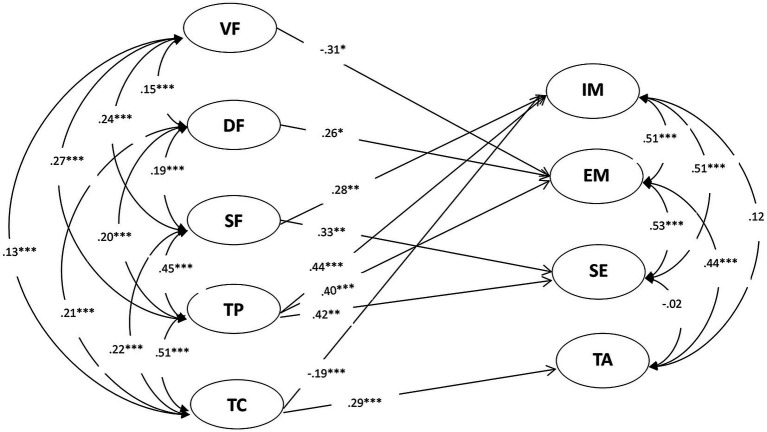
Relationships between female students’ perceptions of teacher feedback and their motivation in language. ^*^p < 0.05, ^**^p < 0.01 and ^***^p < 0.001. VF, verification feedback; DF, directive feedback; SF, scaffolding feedback; TP, teacher praise; TC, teacher criticism; IM, intrinsic motivation; EM, extrinsic motivation; SE, self-efficacy and TA, test anxiety.

As shown in Figure 4, for female students, results suggested that verification feedback had a negative relationship with extrinsic motivation (*r* = −0.31; *p* < 0.05); directive feedback had a positive relationship with intrinsic motivation (*r* = 0.26; *p* < 0.05); scaffolding feedback had positive relationships with intrinsic motivation and self-efficacy (*r*s = 0.28 to.33; *p* < 0.01); teachers’ praise had positive relationships with intrinsic and extrinsic motivation and self-efficacy (*r*s = 0.40 to.44; *p* < 0.001); and teachers’ criticism had a negative relationship with intrinsic motivation (*r* = −0.19; *p* < 0.001) and a positive relationship with test anxiety (*r* = 0.29; *p* < 0.001).

## Discussion

### Gender Differences in Teacher Feedback

Findings suggested that there were no great discrepancies in verification feedback, scaffolding feedback and praise between genders. However, students also perceived that their teachers provided more directive feedback towards female than male students. This may be explained by the fact that female students tended to complete more homework and language practices than their male counterparts; consequently, they may receive more directive feedback from their teachers (Guo, 2017). Moreover, language teachers were perceived by students to provide less criticism to females than males. This may be because females may be generally better in language learning and also tend to study harder (Huang, 2013; Plante et al., 2013), so teachers saw a lesser need to criticize female students on their academic behaviors and achievements.

### Gender Differences in Students’ Motivation

Findings indicated that male and female students’ reported motivation would differ in first language learning. Females reported higher levels of intrinsic and extrinsic motivation, which was consistent with previous research (e.g., Plante et al., 2013; Carr et al., 2016). This finding may explain why female students, compared to male, reportedly show better learning abilities regarding language learning (Huang, 2013; Plante et al., 2013). Additionally, results showed that female students, compared to male, also reported a higher level of test anxiety. This may be because females are naturally expected by their teachers – or parents – to achieve higher scores in language learning, and, together with their higher level of neuroticism, they tended to be more anxious than males (Weisberg et al., 2011).

### Gender Differences in the Relationship Between Teacher Feedback and Student Motivation

More importantly, this study provided insights into gender differences regarding the relationship between teacher feedback and student motivation. There were both similarities as well as distinctions in such relationships. As for similarities, findings indicated that scaffolding feedback and teacher praise had positive correlations with both male and female students’ motivation, paralleling the previous studies (Haimovitz and Corpus, 2011; Guo and Wei, 2019; Guo et al., 2019). This may be because scaffolding feedback serving as an autonomy supportive teaching approach could satisfy students’ needs of competence, autonomy and also relatedness, and thus motivate them in learning (Ryan and Deci, 2000, 2017; Moè and Katz, 2020a). Researchers have suggested that school administers should help increase teachers’ self-compassion which can shape their need satisfaction and increase their willingness to use more autonomy supportive teaching style and provide more scaffolding feedback (Moè and Katz, 2020b).

Additionally, it was also found that teacher criticism had positive correlations with both male and female students’ test anxiety. This finding has a parallel in previous research, which showed that teacher criticism may decrease students’ motivation and increase their test anxiety (Atlas et al., 2004; Guo and Wei, 2019; Thompson et al., 2020).

As for distinctions, first, results suggested that verification feedback showed no significant correlation with males’ motivation while showing a negative correlation with females’ extrinsic motivation. This indicated that females were more sensitive to the grades than males, and that as the amount of verification feedback increased, female students’ extrinsic motivation decreased. This may be because females, compared to males, are generally expected to be better at, and people impose higher expectations over their success in, language learning (Plante et al., 2013); in that regard, when receiving lower grades, female students were more likely to feel depressed and demotivated (Guo, 2017).

Second, findings indicated that directive feedback had a negative correlation with male students’ intrinsic motivation while showing a positive correlation with female students’ extrinsic motivation. This suggested that language teachers’ directive corrections to students’ learning errors/problems may decrease male students’ interest or curiosity while, at the same time, promote female students’ desire to study harder with the intent to gain external rewards, such as recognition from the teacher or parents. This may be because male students are prouder and have higher self-esteems over their own intelligence than female students (Weisberg et al., 2011); therefore, to protect their self-esteem, the first were more resistant to teachers’ directive feedback, which consequently decreased their learning interest. Contrastingly, female students tend to be humbler and have lower self-esteems over their own intelligence (Weisberg et al., 2011); therefore, to improve their scores or please their teachers/parents, they may have more willingly accepted teachers’ directive feedback because they viewed it as a chance for betterment in their academic lives.

Third, findings indicated that teacher criticism had a negative correlation with females’ intrinsic motivation, but the same did not happen for males. This suggested that, for female students, as teachers’ criticism increased, their intrinsic motivation decreased, and that male students were not equally affected. This may be because female students, compared to male, when criticized by the teacher, they may feel more embarrassed, depressed and disappointed (Guo, 2017).

Several limitations should be noted. First, the representativeness of the research sample of this study was limited because participants were obtained only from Shanghai, China. Samples in future-related first language studies should be larger and more representative. Second, this study was based on self-reported measures, and thus, they may be susceptible to response bias. Therefore, future research should employ observational or experimental measures to support the findings of the present study. Third, findings of this study were only from students, and future research may conduct research from the perspective of teachers as well as students to do triangulations for the results. Finally, this study only investigated the role of teacher feedback in student motivation; however, it is also worthwhile to explore how student motivation may affect teachers’ feedback in future study.

## Conclusion

Findings of this study generally indicated that the relationships between students’ perceptions of teacher feedback and students’ reported motivation in first language learning for male and female students would differ. From the *theoretical perspective*, this study contributed to the feedback and motivation literature and may inspire future research in these topics. Further, researchers may need to carefully consider the different roles that teacher feedback plays on male and female students’ motivation in the context of first language learning, a topic that is currently underexplored. Being aware of the impact that this difference plays on students’ motivation is of significance for researchers, school administrators and front-line language teachers because it may allow them to better identify and address the learning needs of individual students (Bandura, 2011; Pereira et al., 2016).

From the *practical perspective*, findings of this study offer important implications for first language teachers who are intent on enhancing their male and female students’ motivation *via* feedback. First, since scaffolding feedback and teachers’ praise had positive correlations with both male and female students’ motivation, teachers should provide the two types of feedback more frequently to increase the latter’s motivation in first language learning settings (Haimovitz and Corpus, 2011; Guo and Wei, 2019; Guo et al., 2019). Second, as our findings generally supported the gender-role stereotyped notion that female students, compared to male, have advantage in first language learning (Yeung et al., 2011; McGeown et al., 2012; D’Lima et al., 2014), it is significant for language teachers to be clearly aware of such stereotypes, and try to challenge them in their feedback. Third, given the negative correlation between verification feedback and females’ extrinsic motivation, first language teachers should try to create a setting in which they can provide less grades, marks or rankings to females (Guo, 2017). Fourth, given the negative correlation between directive feedback and males’ intrinsic motivation and its positive correlation with females’ extrinsic motivation, first language teachers may need to wisely balance the benefits and costs of providing directive feedback (Guo et al., 2019). Finally, teacher criticism showed a negative correlation with female students’ intrinsic motivation. Thus, first language teachers may need to provide less criticism towards females to protect the latter’s interest in learning (Atlas et al., 2004; Guo, 2017; Thompson et al., 2020).

## Data Availability Statement

The raw data supporting the conclusions of this article will be made available by the authors, without undue reservation.

## Author Contributions

WG made the research design, data collection and analysis, and also several rounds of writing and revisions. WZ made contributions in data collection, data analysis, and especially in several round of revisions. Both authors contributed to the article and approved the submitted version.

## Conflict of Interest

The authors declare that the research was conducted in the absence of any commercial or financial relationships that could be construed as a potential conflict of interest.

## Publisher’s Note

All claims expressed in this article are solely those of the authors and do not necessarily represent those of their affiliated organizations, or those of the publisher, the editors and the reviewers. Any product that may be evaluated in this article, or claim that may be made by its manufacturer, is not guaranteed or endorsed by the publisher.
